# Deformable Sensors for Pressure and Position Assessment Using Time-Domain Reflectometry in Motor Rehabilitation

**DOI:** 10.3390/s26154732

**Published:** 2026-07-26

**Authors:** Andrea Cataldo, Antonio Masciullo, Giuseppina Monti, Erika Pittella, Emanuele Piuzzi, Raissa Schiavoni

**Affiliations:** 1 Department of Engineering for Innovation, University of Salento, 73100 Lecce, Italy; antonio.masciullo@unisalento.it (A.M.); giuseppina.monti@unisalento.it (G.M.); 2 Department of Information Engineering, Electronics and Telecommunications, Sapienza University of Rome, 00184 Rome, Italy; erika.pittella@uniroma1.it (E.P.); emanuele.piuzzi@uniroma1.it (E.P.)

**Keywords:** time-domain reflectometry (TDR), deformable sensors, pressure sensing, position sensing, electromagnetic sensing, coaxial sensors, planar sensors, distributed sensing, motor rehabilitation

## Abstract

This work presents the design and preliminary experimental validation of deformable sensors based on time-domain reflectometry (TDR) for rehabilitation-oriented interaction monitoring. Three architectures were investigated: a planar multilayer sensor and two coaxial configurations based on foam and engineered TPU–Hilbert structures. Controlled indentation tests were performed at different positions and deformation levels, extracting two TDR-derived features: the minimum reflection coefficient $\rho_{\min}$, related to deformation intensity, and the perturbation time $t_{\mathrm{pert}}$, related to contact localization. Preliminary calibration curves and two-dimensional maps were used to analyze the coupled dependence of the response on position and indentation depth. Application-oriented manual tests confirmed the different suitability of the three geometries for localized finger pressing, distributed two-hand grasping, and controlled single-hand squeezing. Overall, the results support TDR-based deformable sensors as low-complexity and geometry-adaptable tools for spatially resolved monitoring of motor rehabilitation interactions.

## 1. Introduction

Motor rehabilitation is a fundamental component of clinical practice that aims to restore motor function [1] after neurological [2] or musculoskeletal impairments [3]. It plays a crucial role in improving patients’ quality of life by promoting functional recovery, independence in daily activities, and long-term health outcomes. In this context, objective and quantitative assessment tools are essential to support clinicians in evaluating rehabilitation outcomes [4] and monitoring motor recovery and decline over time [5]. In some neurological rehabilitation contexts, these assessments may also be combined with neurophysiological recordings, such as EEG, to relate motor performance to underlying neural activity [6]. However, many rehabilitation processes still rely on qualitative or semi-quantitative evaluations, which may not fully capture the complexity of motor performance, particularly when fine motor control, force modulation, spatial accuracy, and finger coordination are involved.

A particularly relevant requirement in motor rehabilitation is the possibility of assessing not only whether a patient is able to perform a therapeutic gesture [7], but also how the gesture is performed [8]. This aspect is especially important in upper-limb and hand rehabilitation, where functional recovery depends on the ability to perform controlled and goal-directed interactions with objects and surfaces. Everyday actions such as pressing a button, touching a target, holding an object, opening a container, or manipulating tools require the combined control of contact location, applied force, and finger coordination. Therefore, the quantitative evaluation of these components can provide useful information on residual impairment, compensatory strategies, and the effectiveness of the rehabilitation program. Different rehabilitation tasks emphasize these motor components in different ways. Differential and task-oriented rehabilitation approaches have been investigated to promote adaptable motor recovery [9], whereas unilateral and bilateral upper-limb task training has been evaluated in post-stroke rehabilitation [10]. Interactive finger–hand rehabilitation exercises have also been proposed [11], together with repetitive training of isolated hand movements [12]. Localized pressing exercises mainly assess selective finger control and spatial accuracy, since the patient is required to apply pressure on a specific target region while avoiding unintended contact elsewhere [13]. Fine-motor and finger-coordination tasks [14] focus on the controlled activation of one or more fingers and are useful for evaluating precise, goal-directed movements [15] and coordinated finger control [16]. Distributed contact tasks [17] involve a larger portion of the hand, for example during interaction with compliant pads, extended supports, or deformable surfaces, where the spatial distribution of the applied load may be as relevant as its overall magnitude. Finally, grasping and squeezing tasks [18] are closely related to functional hand use, because they require circumferential contact with an object and grip-force modulation, as investigated in force-feedback robotic rehabilitation [19]. These tasks also require coordinated multi-finger and multi-joint action [20].

**Figure 1:**
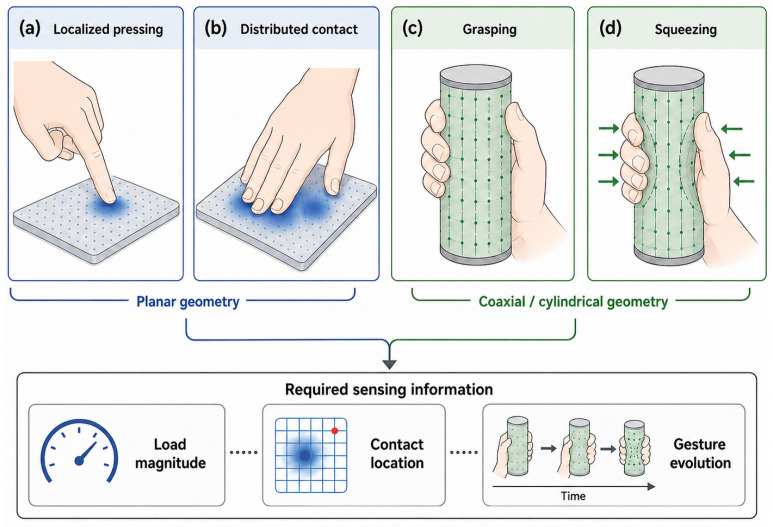
Schematic overview of the rehabilitation-inspired interaction modalities considered in this work: (**a**) localized pressing, (**b**) distributed contact, (**c**) grasping, and (**d**) squeezing. Localized pressing and distributed contact are associated with planar sensing geometries, whereas grasping and squeezing motivate coaxial or cylindrical configurations. These modalities require information on load magnitude, contact location, and gesture evolution.

From a sensing perspective, these rehabilitation tasks require monitoring systems able to capture not only the occurrence of contact, but also its intensity, position, and temporal evolution. This is important because inaccurate contact, inappropriate force levels, or poorly controlled multi-finger interaction may reduce exercise effectiveness and promote compensatory motor patterns [21]. This is not a trivial requirement, because the physical interaction between the patient and the rehabilitation device can vary substantially depending on the motor task. Flat or extended sensing surfaces are suitable for localized pressing and distributed contact, whereas grasping and squeezing require structures that can conform to non-planar or cylindrical objects. Accordingly, the sensor should be mechanically compliant, spatially informative, and adaptable to different interaction geometries. This motivates the development of deformable sensors capable of providing force- and position-related information across different rehabilitation tasks, while preserving the same sensing principle in different geometrical configurations. Figure 1 provides a schematic overview of the rehabilitation-inspired interaction modalities considered in this work and highlights their relationship with the required sensing information and sensor geometries.

In this context, Time-Domain Reflectometry (TDR) [22] is particularly attractive because it enables distributed sensing through the analysis of reflected electromagnetic signals. TDR has been widely used for non-destructive monitoring and characterization in several fields. Microwave reflectometric systems have been proposed for distributed monitoring applications [23], while embedded sensing structures have been investigated for structural health monitoring [24]. TDR-based approaches have also been developed for monitoring structural phenomena such as bridge scour [25]. In geotechnical and environmental applications, TDR has been investigated for soil characterization [26], water-level monitoring [27], continuous monitoring of nitrate in soil and water [28], and the extraction of dielectric parameters for monitoring watered and contaminated soils [29]. Biomedical and material-related applications have also been explored [30], mainly because local impedance variations can be detected through a relatively simple measurement configuration. In a TDR-based measurement, an electromagnetic signal is launched along a sensing structure, and local impedance variations generate reflected perturbations that can be observed in the time-domain reflection response. Since the proposed sensors are directly interrogated as planar or coaxial transmission-line structures, their response is not dependent on an externally imposed electromagnetic polarization. The electric-field distribution is instead determined by the conductor geometry and dielectric configuration of the sensing structure.

When a mechanical deformation locally modifies the conductor spacing and dielectric distribution, and consequently the local impedance of the sensing structure, a corresponding perturbation appears in the time-domain reflection coefficient. The amplitude of this perturbation can provide information related to the intensity of the mechanical interaction, while its temporal location can be associated with the position at which the interaction occurred along the sensing path. Thus, TDR offers the possibility of extracting pressure- and position-related descriptors without requiring a dense array of independent sensing elements. This characteristic is particularly suitable for deformable rehabilitation-oriented sensors, because the same electromagnetic sensing principle can be adapted to different mechanical layouts while preserving a relatively simple measurement framework.

Building on this concept, this work investigates deformable TDR-based sensors for rehabilitation-oriented pressure and position monitoring. Rather than proposing a single optimized sensor shape, the study evaluates whether the same TDR-based sensing strategy can be implemented in complementary deformable architectures tailored to different interaction modalities. Specifically, three configurations are considered: a planar multilayer sensor intended for localized pressing and distributed contact over an extended surface, a foam-based coaxial sensor intended for compliant grasping and broader circumferential interaction, and a TPU–Hilbert coaxial sensor designed to explore a more engineered and repeatable response during controlled squeezing. The three configurations are first characterized under controlled loading conditions, considering different indentation levels and load positions. These measurements are used to derive calibration relationships and spatial response maps, providing a quantitative description of the sensor response to deformation magnitude and contact location. Subsequently, preliminary measurements during representative manual gestures are performed to verify whether the trends observed under controlled conditions are preserved in more realistic interaction scenarios.

The paper is organized as follows. Section 2 reviews existing rehabilitation sensing technologies and outlines the addressed technological gap. Section 3 describes the developed sensor architectures, while Section 4 presents the experimental setup, testing protocols, and data-analysis procedures. Section 5 reports the experimental results, Section 6 discusses the main findings and limitations, and Section 7 summarizes the conclusions and future work.

## 2. State of the Art and Rationale of the Work

Several sensing technologies have been proposed to support quantitative assessment in motor rehabilitation. Sensorized gloves represent one of the most common solutions for hand and finger monitoring. Haptic glove systems combined with semi-immersive virtual reality have been investigated for upper-extremity motor rehabilitation after stroke [31]. Wearable systems based on soft gloves have also been developed for hand rehabilitation [32], including portable assistive gloves intended for stroke survivors with severe hand impairment [33] and wearable rehabilitation gloves incorporating compliant actuators [34]. These systems can integrate bending, inertial, tactile, or force sensors directly on the hand and are particularly useful for monitoring finger motion, grasping patterns, or hand-object interaction during functional tasks. However, glove-based systems may be expensive, may require complex calibration, and may interfere with natural hand movements, especially when several sensors, cables, or actuation components are integrated into the wearable structure. Moreover, they are not always suitable when the therapeutic task requires interaction with an external deformable surface or object rather than direct instrumentation of the patient’s hand.

Soft robotic systems and smart robotic gloves [35] are increasingly used in rehabilitation for hand assistance, motor training, and haptic or force feedback. By combining compliant actuation and embedded sensing, they can support assisted or guided movements during rehabilitation tasks. However, their use often requires actuators, control units, sensors, and calibration procedures, increasing system complexity, cost, and maintenance. Therefore, when the objective is mainly to monitor the interaction with an external deformable object or surface, simpler and more easily reconfigurable sensing structures may be preferable.

Force platforms and pressure platforms are another widely used class of devices for rehabilitation and biomechanical assessment. Platform-based robot-assisted rehabilitation has been systematically investigated for musculoskeletal and neurological conditions [36], while calibration methods and devices have been developed for force platforms used in medical applications [37]. These systems provide reliable information about global loads, weight distribution, and postural control. However, they are generally optimized for global or planar force assessment and are less suited to resolving localized finger-level interactions, multi-point contact, or grasping actions around non-planar objects. Similarly, vision-based systems can reconstruct movement trajectories and provide valuable kinematic information. Vision-based serious games and virtual-reality systems have been reviewed in the context of motor rehabilitation [38], while mirror-feedback strategies have been proposed to improve interactive rehabilitation systems for users with disabilities [39]. More broadly, computer-vision-based approaches have been investigated for physical rehabilitation and assessment [40]. However, vision-based systems require controlled acquisition conditions, are sensitive to occlusions, and cannot directly measure the applied force. Therefore, although vision systems are useful for motion analysis, they are often insufficient when the therapeutic outcome depends on the magnitude and localization of contact forces.

On the other hand, flexible tactile sensors, including resistive, capacitive, piezoresistive, and optical solutions, have also been investigated for wearable and rehabilitation-oriented applications [41]. These technologies can be integrated into soft surfaces and can provide local information about contact pressure. Nevertheless, they often require arrays of sensing elements to achieve spatial resolution, increasing the number of electrical connections, acquisition channels, and calibration procedures. This can become particularly limiting when the objective is to monitor distributed interactions over elongated or deformable structures, or when the sensing geometry must be adapted to different rehabilitation tasks.

Fiber-optic sensing technologies, including fiber Bragg grating (FBG)-based systems, have also been explored in biomechanics and rehabilitation because of their high sensitivity, compact sensing elements, and immunity to electromagnetic interference. Fiber-optic force sensors have been reviewed for interventional and rehabilitation applications [42], while wearable optical-fiber sensors have been specifically investigated for rehabilitation monitoring [43]. In particular, multiple FBG sensing elements can be multiplexed along the same optical fiber, allowing deformation- or load-related information to be associated with predefined grating locations and thus enabling spatially resolved measurements. However, the available sensing locations and their spatial distribution are determined by the number and placement of the gratings. Moreover, FBG-based systems generally require wavelength-selective optical interrogation units, appropriate mechanical transduction structures, and careful packaging and integration, which may increase complexity and cost when different deformable geometries or interaction modalities are considered.

**Figure 2:**
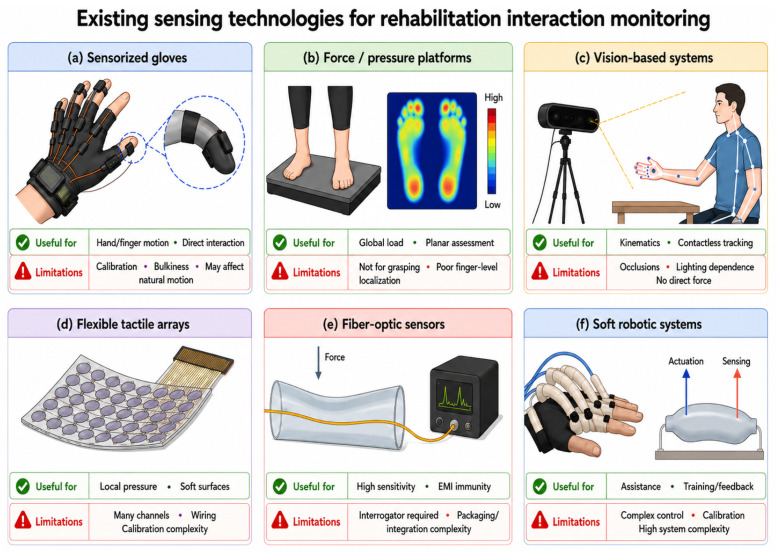
Schematic overview of existing sensing technologies for rehabilitation interaction monitoring. These technologies provide valuable information for motor assessment while differing in terms of measured quantities, spatial sensing strategy, calibration, wearability, interaction geometry, wiring, interrogation requirements, integration complexity, actuation/control requirements, and system cost.

Figure 2 provides a schematic overview of the main sensing technologies discussed in this section, including sensorized gloves, soft robotic systems, force/pressure platforms, vision-based systems, flexible tactile arrays, and fiber-optic sensors. The figure summarizes their typical use in rehabilitation monitoring together with their main practical advantages and limitations.

Taken together, these approaches provide valuable and complementary tools for rehabilitation assessment. Sensorized gloves are effective for direct hand instrumentation but may interfere with natural movement and are not always suitable for monitoring interaction with external deformable surfaces or objects. Soft robotic systems can provide assistance and feedback, but they often involve actuators, control units, and calibration procedures that increase system complexity. Force and pressure platforms provide reliable global or planar measurements, but are less suited to non-planar grasping and localized finger-level contact. Vision-based systems capture kinematics but do not directly measure contact force. Flexible tactile arrays can provide spatially resolved pressure information through multiple sensing cells, whereas multiplexed FBG systems provide localized deformation- or load-related information at predefined grating positions. However, these solutions may require task-specific sensing layouts, multiple acquisition channels, dedicated interrogation units, or mechanically tailored integration strategies. Consequently, the most appropriate sensing technology depends on the target rehabilitation gesture, the interaction geometry, the required spatial resolution, and the acceptable level of system complexity.

Within this context, the present work investigates deformable TDR-based sensing structures that can be adapted to the target motor task while maintaining a common electrical interrogation principle. In the proposed approach, position-related information is extracted from the temporal location of a perturbation produced along a deformable transmission-line structure, while the perturbation amplitude provides information mainly related to deformation intensity. This principle is investigated through planar and coaxial architectures designed for different rehabilitation-inspired interaction modalities. The aim is not to replace established sensing technologies or to claim superior localization performance, but to explore a relatively low-complexity and geometry-adaptable approach for extracting position- and intensity-related information from deformation-induced changes in the reflected response.

The following sections describe the developed sensor architectures and the experimental procedures used to assess their response under controlled and rehabilitation-inspired interaction conditions.

## 3. Developed Deformable Sensor Architectures

The three deformable sensor architectures were developed to investigate the adaptability of the proposed TDR-based sensing approach to different rehabilitation-oriented interaction modalities. The first configuration is a planar multilayer sensor intended for localized pressing and distributed contact over an extended surface, whereas the other two are coaxial sensors designed for circumferential interaction, grasping, and squeezing tasks. The two coaxial devices share the same overall cylindrical sensing geometry but differ in their internal dielectric structure, namely an expanded polyurethane foam and a 3D-printed TPU–Hilbert lattice. The dimensions of each architecture were selected by considering the intended interaction modality, fabrication constraints, and the need to obtain a measurable deformation-induced variation in the TDR response. For the coaxial configurations, the conductor dimensions and their relative spacing were also chosen to maintain a suitable transmission-line geometry while allowing the structure to be comfortably grasped and sufficiently deformed under manual loading. Excessively large diameters were avoided, since they could reduce the relative change in conductor spacing produced by a given compression and increase the force required to obtain a measurable response. The resulting dimensions therefore represent application-oriented prototype choices rather than the outcome of a formal geometric or electromechanical optimization. The following subsections describe the design rationale, materials, dimensions, and structural characteristics of each configuration.

### 3.1. Planar Sensor

**Figure 3:**
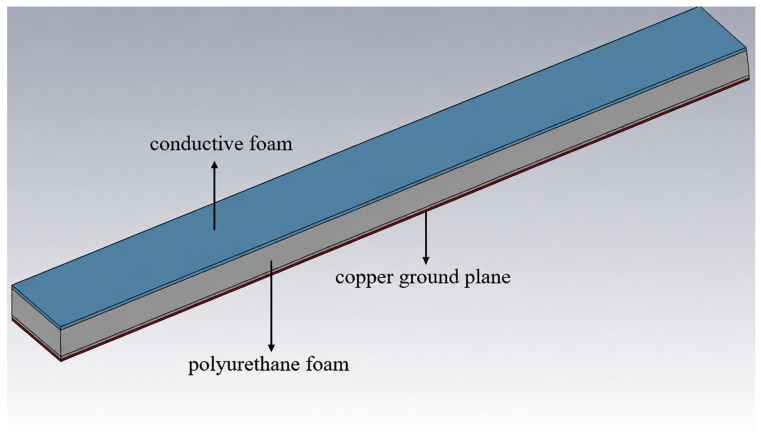
TDR planar sensor architecture with conductive foam, polyurethane dielectric, and copper ground plane.

The first developed configuration is based on a planar soft sensing structure and represents a deformable sensor designed for distributed pressure and position sensing in rehabilitation applications. It is shown in Figure 3.

The planar sensor was developed as a multilayer deformable structure designed to combine mechanical compliance with distributed electromagnetic sensing. The device consists of a continuous sensing surface in which compliant dielectric and conductive layers are arranged to support guided-wave propagation and to make the electromagnetic response sensitive to local mechanical deformation. The lower layer is a copper ground plane, which provides the electromagnetic reference for the guided structure. Above it, a compressible dielectric layer made of open-cell polyurethane foam, with a nominal thickness of ∼1cm, acts as the main compliant substrate. This material exhibits a relatively low dielectric permittivity ($\varepsilon_{r}$ = 1.24–1.45) and low loss tangent (tanδ = 0.015–0.03), enabling quasi-TEM propagation while limiting signal attenuation. Its high compressibility and elastic recovery allow the structure to deform under applied loads and return to its original shape after unloading, which is essential for repeated use in rehabilitation-oriented applications. The uppermost layer is a conductive foam, with a thickness of approximately 1–2 mm, an effective dielectric permittivity of $\varepsilon_{r}$ = 2–3, and a relatively high loss tangent (tanδ = 0.1–0.2). This layer contributes to the overall sensing structure together with the compressible dielectric layer and the copper ground plane. When an external load is applied, the resulting local deformation modifies the impedance distribution of the multilayer sensing path and, consequently, the reflected response in the time domain. Owing to this continuous layered architecture, the planar sensor can provide deformation- and position-related information without relying on discrete sensing elements, while preserving mechanical compliance and recovery under repeated loading.

### 3.2. Coaxial Sensors

**Figure 4:**
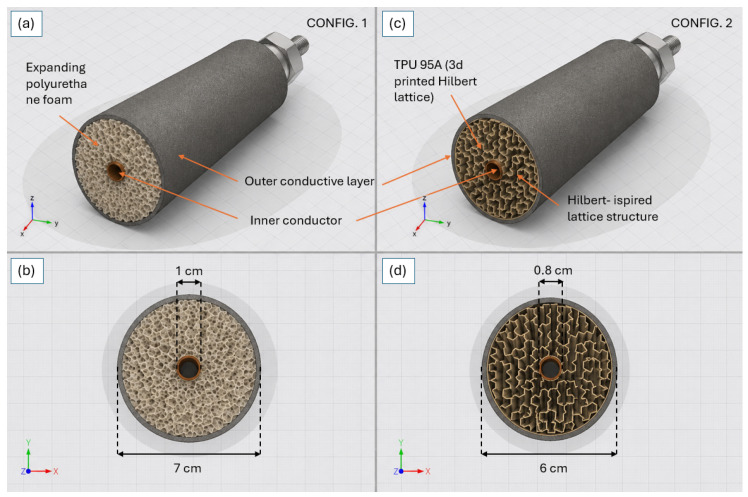
Coaxial sensor configurations developed for circumferential interaction, grasping, and squeezing tasks: (**a**) 3D view of Config. 1, based on an expanding polyurethane foam dielectric; (**b**) front cross-sectional view of Config. 1; (**c**) 3D view of Config. 2, based on a TPU 95A dielectric with a 3D-printed Hilbert-inspired lattice structure; (**d**) front cross-sectional view of Config. 2. Both configurations preserve the same coaxial sensing principle while implementing different dielectric architectures.

The coaxial sensor configurations have been developed to extend the sensing capability of the proposed approach to interaction modalities involving circumferential contact, grasping, and squeezing actions, which cannot be effectively captured using planar structures. In contrast to the planar sensor, the coaxial geometry enables distributed sensing over a closed cylindrical surface, making it particularly suitable for applications in which the user interacts with the device through enclosing or multi-finger contact. As shown in Figure 4, the two implemented coaxial sensors share the same overall cylindrical geometry while differing in the internal dielectric configuration.

Among the considered configurations, a first coaxial sensor has been realized using a deformable bulk dielectric medium obtained from a two-component expanding polyurethane foam, as illustrated in Figure 4a,b. The dielectric is produced by mixing two liquid components (base and activator) in a predefined weight ratio, triggering a chemical reaction that leads to volumetric expansion and the formation of a lightweight porous structure. The resulting material is characterized by a low effective density approximately 55–90 $\mathrm{kg}/m^{3}$ and a highly compliant mechanical behavior due to the presence of randomly distributed air-filled cells. The sensor is shaped into a cylindrical geometry with a radius of approximately 3.5 cm and a length of 22 cm. This configuration provides several advantages: the expansion process naturally fills the available volume, ensuring good mechanical continuity and conformability, while the intrinsic compressibility of the foam enables large deformations under relatively low applied forces. From a sensing perspective, the random porous structure allows distributed impedance variations along the sensor, making it suitable for capturing general interaction patterns without requiring complex fabrication processes. At the same time, the stochastic nature of the internal geometry may introduce variability in the local electromagnetic response, particularly in terms of spatial resolution and repeatability.

To address these aspects while maintaining mechanical flexibility, a second coaxial sensor has been developed based on an engineered dielectric structure, as shown in Figure 4c,d. In this case, thermoplastic polyurethane (TPU 95A) is employed as the base material due to its combination of elasticity, mechanical robustness, and compatibility with additive manufacturing. The material exhibits a high elongation at break (up to several hundred percent) and a Shore A hardness of 95, allowing significant deformation while preserving structural integrity over repeated loading cycles. The dielectric is fabricated using a 3D printing process, enabling precise control over the internal geometry of the sensor.

**Figure 5:**
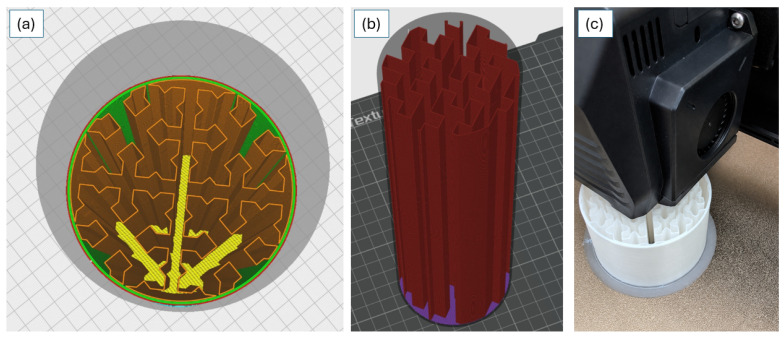
Engineered dielectric structure and fabrication view of the TPU-based coaxial sensor: (**a**) top view of the internal lattice geometry based on a Hilbert-inspired space-filling pattern; (**b**) three-dimensional view of the cylindrical structure, highlighting the vertically extruded architecture; (**c**) photograph of the actual TPU–Hilbert structure during additive manufacturing, showing the fabricated internal lattice morphology.

As can be observed in Figure 5, the resulting structure is cylindrical, with a radius of approximately 3 cm and a length of 20 cm, and incorporates an internal lattice designed according to a space-filling pattern inspired by the Hilbert curve. This geometry maximizes the internal surface area within a confined volume and creates a highly interconnected network of deformable elements. Unlike the bulk foam configuration, this engineered structure allows the mechanical response to be guided through predefined deformation pathways, improving the uniformity and predictability of the interaction. From an electromagnetic standpoint, this translates into a more controlled variation in the local impedance along the sensor, potentially enhancing spatial discrimination and measurement repeatability. At the same time, the structured design maintains sufficient compliance to support grasping and squeezing actions typical of rehabilitation tasks. Both coaxial sensors operate according to the same TDR-based sensing principle, in which mechanical deformation induces local impedance variations that are detected through changes in the time-domain reflection coefficient ρ(t). The two configurations should therefore be interpreted as complementary design strategies: the first provides a simple, low-cost, and highly compliant solution that is easy to fabricate and suitable for general-purpose sensing, while the second demonstrates how structural engineering can be leveraged to achieve a more controlled and tunable sensing response. This comparison highlights the flexibility of the proposed approach, showing that different sensor designs can be tailored to specific application requirements without modifying the underlying sensing methodology.

## 4. Experimental Setup and Testing Methods

### 4.1. Measurement Setup

**Figure 6:**
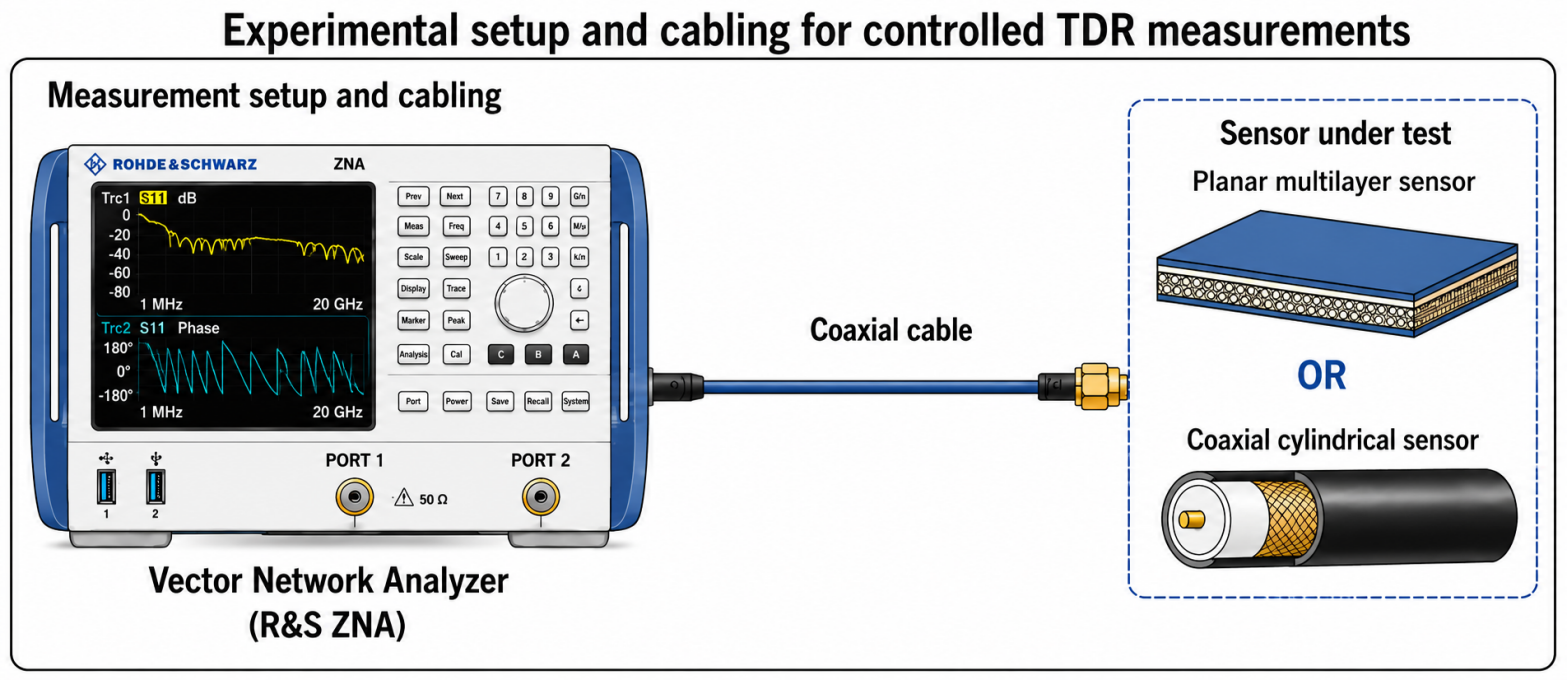
Schematic overview of the measurement setup, comprising the Rohde & Schwarz ZNA vector network analyzer, the coaxial cable, the direct SMA connection, and the sensor under test. The complex reflection coefficient $S_{11}\left(f\right)$ was measured in the frequency domain and transformed into the corresponding time-domain response ρ(t) through an inverse Fourier transform.

All measurements were performed using a Rohde & Schwarz ZNA vector network analyzer (VNA) operating in one-port reflection mode. Each sensor was connected to the VNA through a coaxial cable and an SMA connection. Before each measurement session, a calibration procedure was performed at the connector plane to minimize the contribution of the cable and reference the measurements to the sensor input. The instrument measured the magnitude and phase of the complex reflection coefficient $S_{11}\left(f\right)$ over the selected frequency range. The corresponding time-domain response ρ(t) was then obtained by applying an inverse Fourier transform to the measured frequency-domain data. The resulting reflectograms were used to analyze the deformation-induced impedance perturbations along the sensing structure. In particular, variations in the amplitude of the reflected response were associated with the magnitude of the mechanical deformation, whereas shifts in the temporal position of the perturbation were related to the location of the applied load. Figure 6 provides a schematic overview of the measurement chain and sensor connection.

The acquired time-domain data were exported and subsequently processed and visualized in MATLAB R2025a.

### 4.2. Controlled Mechanical Testing

All tests were performed according to a controlled and repeatable protocol aimed at characterizing the electromechanical response of the proposed sensors under different load positions and indentation levels. The same mechanical loading system was used for both planar and coaxial configurations in order to ensure comparability among the different sensing architectures.

**Figure 7:**
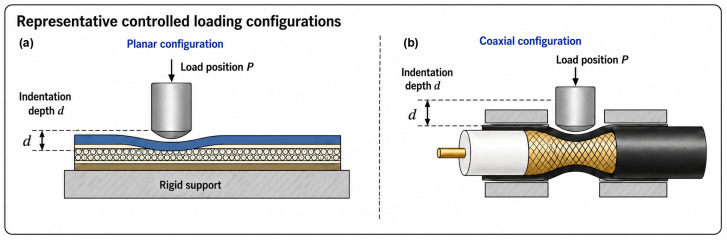
Schematic representation of the controlled loading configurations adopted for the two sensor architectures: (**a**) planar sensor and (**b**) coaxial sensor. For each configuration, the indentation depth *d* was imposed at selected load positions *P*.

**Figure 8:**
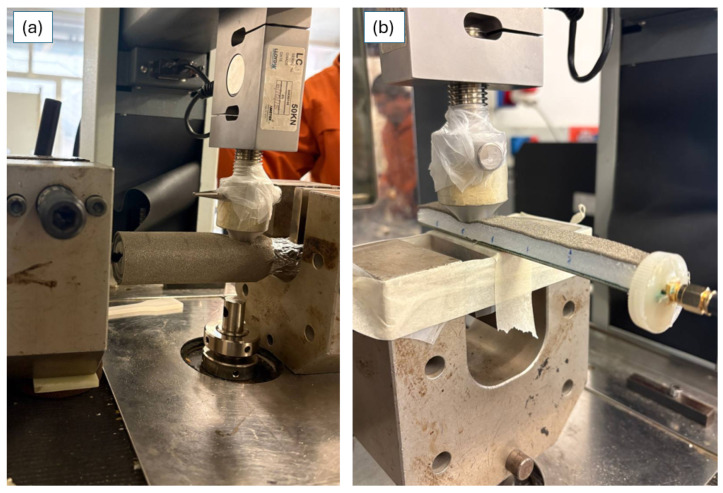
Photographs of the mechanical loading setup used during the experiments: (**a**) controlled indentation test on a coaxial sensor and (**b**) controlled indentation test on the planar sensor. In both configurations, the sensor was positioned on an electrically insulating PTFE support, while a calibrated vertical translation stage applied controlled indentation at selected positions.

Figure 7 schematically illustrates the loading principle adopted for the two sensor geometries. In both configurations, a localized vertical displacement was applied at selected load positions *P*, while the imposed indentation depth *d* was systematically varied. The schematic provides a simplified representation of the loading conditions, whereas the corresponding laboratory implementations are shown in Figure 8.

In the actual experimental setup, each sensor was positioned on an electrically insulating PTFE support to avoid unwanted capacitive or conductive coupling with the laboratory bench. A calibrated vertical translation stage was used to apply the prescribed indentation depths at the selected positions along the sensing structure.

For the planar sensor, the upper surface of the sensor was marked with five spatial references, labelled from P1 to P5, and the mechanical load was applied sequentially at each position. For each spatial point, the indentation depth was adjusted in discrete steps of 3 mm, 4 mm, 5 mm, 6 mm, and 7 mm, each corresponding to a different applied normal force. For the coaxial sensors, the same mechanical loading setup and acquisition procedure were adopted, but the spatial references and indentation range were adapted to the cylindrical geometry and mechanical compliance of the devices. The first coaxial configuration, based on the expanding polyurethane foam dielectric, was tested at six spatial positions, labelled from P1 to P6, whereas the TPU-based configuration with Hilbert-inspired lattice was tested at four spatial positions, labelled from P1 to P4. At each selected position, the compressive interaction was applied with predefined indentation depths of 3 mm, 5 mm, 7 mm, 10 mm, 13 mm, and 15 mm. These values were selected to investigate the response of the coaxial sensors over a wider compression range, compatible with grasping and squeezing actions. At each position–indentation combination, the loading sequence was performed in a controlled manner. The indenter was lowered until the desired displacement was reached, after which a stabilization interval was allowed to reduce transient mechanical effects and promote repeatable contact conditions. The sensor was then excited through Port 1 of the VNA, and the corresponding time-domain reflection profile ρ(t) was acquired using the internal transformation of the measured $S_{11}\left(f\right)$. Once the full set of indentation levels had been completed at one spatial position, the loading system was translated to the next position, and the same sequence was repeated. This protocol was designed to evaluate two complementary aspects of the proposed sensors: first, their sensitivity to different applied loads at a fixed position, assessed through variations in the amplitude of the reflected perturbation; and second, their ability to identify the location of the mechanical interaction, assessed through the temporal shift in the reflection response as the load position was varied.

### 4.3. Manual Interaction Testing

**Figure 9:**
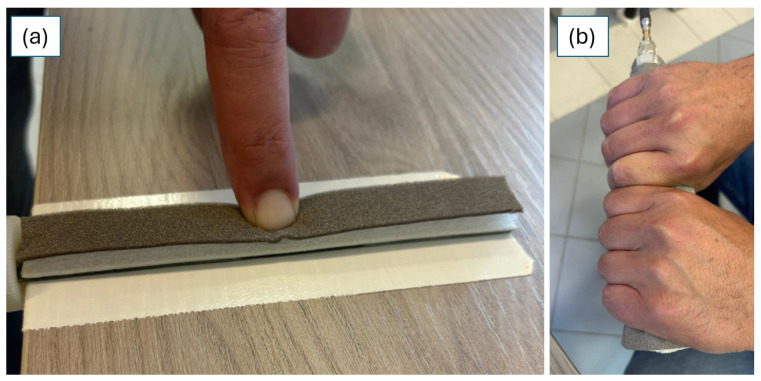
Manual interaction tests performed on the proposed sensors: (**a**) localized finger pressing on the planar sensor at a selected contact position; (**b**) bimanual grasping of the foam-based coaxial sensor, representative of distributed circumferential interaction.

Preliminary manual interaction tests were also performed to assess the response of the sensors under more realistic rehabilitation-inspired gestures. These tests were used as application-oriented demonstrations of the sensing behavior observed during controlled mechanical loading. For the planar sensor, localized finger-pressing tests were performed at predefined positions along the sensing structure. At each position, the user applied the maximum comfortable pressure with one finger, and the corresponding TDR response was acquired. This test was aimed at evaluating the suitability of the planar configuration for point-like pressing tasks and contact localization. For the coaxial sensors, grasping tests were performed to evaluate their response to circumferential interactions. The TPU–Hilbert coaxial sensor was tested during a single-hand loading–unloading sequence, in which the device was progressively squeezed and then gradually released. This test allowed the observation of the TDR response during both increasing grip intensity and signal recovery. The foam-based coaxial sensor was instead tested under larger and more distributed grasping conditions, including bimanual interaction, consistent with its larger size and compliant structure. These manual tests complement the controlled indentation experiments by showing that the proposed TDR-based sensing approach can be adapted to different interaction modalities. The manual interaction configurations considered in this study are shown in Figure 9.

### 4.4. Feature Extraction and Calibration-Map Construction

In addition to the qualitative analysis of the time-domain reflectograms, a quantitative feature-based analysis was performed to describe the response of each sensor under controlled mechanical loading. The objective of this analysis was to provide a first quantitative characterization of the sensor response by extracting simple and physically interpretable descriptors related to the two controlled mechanical variables imposed during the experiments, namely the load position (*P*) and the indentation depth (*d*). For each sensor, the reflection coefficient ρ(t) was analyzed within a selected time window corresponding to the deformation-induced perturbation. This region of interest (ROI) was manually identified from the measured reflectograms so as to include the portion of the signal most affected by the applied mechanical load. The same extraction criterion was then applied consistently to all measurements belonging to the same sensor, allowing the extracted descriptors to be compared across the different position–indentation combinations.

Two main features were considered. The first one was the minimum value of the reflection coefficient within the selected ROI:

(1) $$\rho_{\min}\left(P,d\right)=\min_{t\in \mathrm{ROI}}\rho \left(t;P,d\right)$$

where *P* denotes the load position and *d* the imposed indentation depth. This descriptor was selected as a simple amplitude-related feature because, in the considered measurements, the mechanical deformation produced a local dip or reduction in the reflection profile. Therefore, $\rho_{\min}$ provides an easily interpretable indicator of the deformation-induced perturbation: lower values correspond to a more pronounced modification of the TDR response and can be associated with stronger mechanical interaction under controlled conditions. The second extracted feature was the temporal position of the perturbation, denoted as $t_{\mathrm{pert}}$. This parameter was identified as the time instant corresponding to the selected local feature within the same ROI, for example the time at which the local minimum occurs:

(2) $$t_{\mathrm{pert}}\left(P,d\right)=\arg\min_{t\in \mathrm{ROI}}\rho \left(t;P,d\right)$$

This descriptor was used to evaluate the spatial localization capability of the sensor, since perturbations occurring farther from the excitation port are expected to appear later in the time-domain response according to the TDR principle. Thus, $\rho_{\min}$ and $t_{\mathrm{pert}}$ provide complementary information: the former is mainly related to the magnitude of local deformation, whereas the latter is mainly associated with the position of the mechanical interaction. Nevertheless, these descriptors cannot be considered fully independent, since both the amplitude and temporal responses may be affected by the combined influence of load position and indentation depth.

As a first representation, one-dimensional feature-response curves were extracted under selected conditions. In particular, $\rho_{\min}$ was plotted as a function of indentation depth at fixed load positions to highlight the deformation sensitivity of each sensor. Conversely, $t_{\mathrm{pert}}$ was plotted as a function of load position at fixed indentation depths to assess the localization capability of the TDR response. In particular, polynomial fits were used as empirical calibration-oriented representations of the observed trends under selected testing conditions. These fits provide a compact way to describe the relationship between the extracted features and the controlled mechanical variables, supporting the comparison among sensor architectures and the identification of trends relevant for future calibration procedures.

However, to obtain a broader view of the response, the feature extraction was then extended to all available position–indentation combinations acquired during the controlled mechanical tests. For each sensor, the extracted values were arranged into two-dimensional feature maps, where columns correspond to the load positions and rows correspond to the imposed indentation depths. In this representation, the amplitude-related and temporal responses can be expressed as:

(3) $$\rho_{\min}=f\left(P,d\right)$$

(4) $$t_{\mathrm{pert}}=g\left(P,d\right)$$

These maps were used as preliminary feature-based representations of the sensor response under controlled loading conditions. The $\rho_{\min}$ map allows the dependence of the amplitude-related response on both deformation intensity and contact location to be visualized, whereas the $t_{\mathrm{pert}}$ map describes how the temporal position of the reflected perturbation changes across the sensing structure and with the imposed deformation condition. Rather than serving as final calibration models, the two-dimensional maps provide a compact way to analyze the coupled effect of position and indentation depth on the TDR response. This representation is useful to verify whether the measured trends are consistent with the expected sensing behavior, to compare the three developed sensor architectures, and to identify the conditions under which future, more systematic calibration procedures could be defined through repeated and dedicated measurements. Overall, the proposed feature extraction and mapping procedure provides a quantitative framework for interpreting the controlled mechanical tests. The one-dimensional curves highlight selected deformation- and localization-related trends, while the two-dimensional maps provide a more complete overview of the response over the tested position–indentation space. This analysis also provides a reference for interpreting the manual interaction tests discussed in the following section.

## 5. Experimental Results

### 5.1. Controlled Mechanical Characterization

The experimental response of the developed sensors was analyzed with respect to the two main sensing functions required for rehabilitation-oriented monitoring: discrimination of the applied compression level and localization of the mechanical interaction along the sensing structure. Accordingly, two complementary controlled conditions were considered. First, the load position was kept fixed while the indentation depth was varied, in order to evaluate the sensitivity to compression. Second, the indentation depth was kept fixed while the load position was varied, in order to assess the spatial encoding capability of the TDR response. For clarity, representative profiles are reported in the main text, although the same analysis was performed for all tested position–indentation combinations and for each sensor architecture.

**Figure 10:**
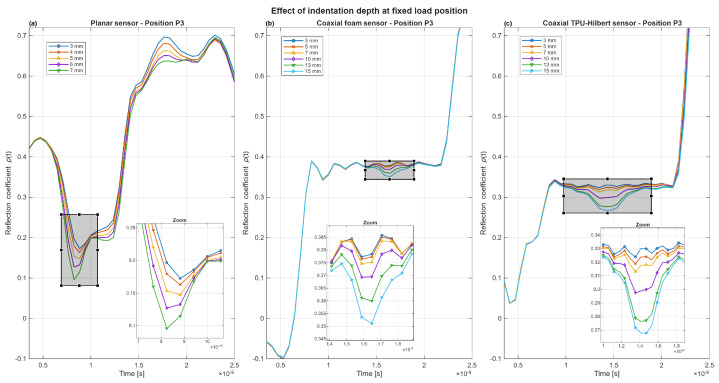
Effect of indentation depth at a fixed load position for the three sensor architectures: (**a**) planar sensor at P3, (**b**) coaxial sensor with expanded polyurethane foam dielectric at P3, and (**c**) TPU-based coaxial sensor with Hilbert-inspired lattice at P3. The indentation range is 3–7 mm for the planar configuration and 3–15 mm for the coaxial configurations. Insets highlight the local perturbation region used to assess the amplitude modulation induced by increasing compression.

Figure 10 shows the time-domain reflection coefficient ρ(t) measured at the representative load position P3 for different indentation depths. The three panels compare the planar sensor, the coaxial sensor based on expanded polyurethane foam, and the TPU-based coaxial sensor with Hilbert-inspired internal lattice. The indentation range differs according to the mechanical protocol adopted for each geometry: 3–7 mm for the planar sensor and 3–15 mm for the coaxial sensors.

As shown in Figure 10a, the planar sensor exhibits a clear modulation of the reflection profile as the indentation depth increases. The most relevant variation occurs in the highlighted perturbation region, where the negative reflection feature becomes progressively more pronounced for increasing indentation. This behavior indicates that the applied load modifies the local characteristic impedance of the multilayer structure, mainly through changes in the mechanical coupling between the conductive foam layer, the compressible dielectric layer, and the copper ground plane. The marked amplitude modulation confirms the suitability of the planar architecture for detecting localized pressing actions over an extended surface. For the coaxial sensor based on expanded polyurethane foam, reported in Figure 10b, increasing indentation also produces a detectable modification of the local reflection feature, although the amplitude variation is less pronounced than in the planar case. This behavior can be attributed to the highly compliant and randomly porous structure of the bulk foam dielectric, which distributes the mechanical deformation over an irregular internal cellular network. As a result, the load-induced impedance perturbation is smoother and less localized, leading to a more limited amplitude excursion. In contrast, the TPU-based coaxial sensor with Hilbert-inspired lattice, shown in Figure 10c, exhibits a more evident amplitude modulation within the perturbed region. The deeper negative feature suggests that the engineered internal geometry improves the electromechanical transduction of the applied compression. Unlike the random porous foam, the Hilbert-inspired lattice introduces predefined deformation pathways and a more structured internal dielectric distribution, leading to a more localized and repeatable impedance variation in the time-domain response.

The complementary localization capability was evaluated by keeping the indentation depth fixed and varying the load position along each sensor. In this case, the relevant information is mainly associated with the temporal displacement of the reflected perturbation rather than with its amplitude.

**Figure 11:**
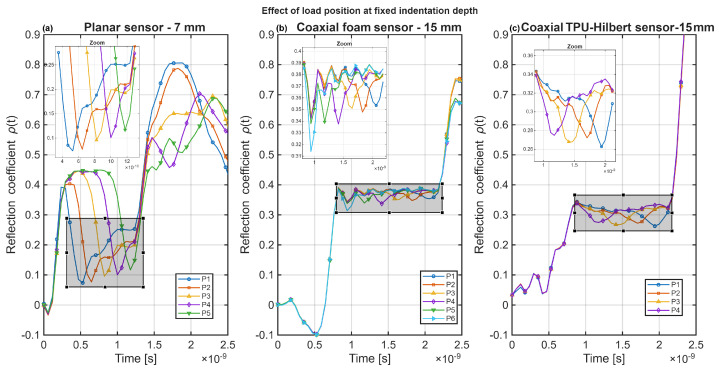
Effect of load position at a fixed indentation depth for the three sensor architectures: (**a**) planar sensor at 7 mm, (**b**) foam-based coaxial sensor at 15 mm, and (**c**) TPU–Hilbert coaxial sensor at 15 mm. The curves correspond to different load positions, while the insets highlight the resulting temporal shifts in the local reflection perturbation.

Figure 11 shows that, at fixed indentation depth, the local reflection perturbation shifts in time as the load position changes. This behavior is consistent with the TDR principle, since a mechanical discontinuity located farther from the feeding point produces a reflected perturbation at a later time. The observed temporal displacement confirms that the contact location is encoded in the time-domain response for all tested architectures. Therefore, the proposed sensors are able to provide not only information on the occurrence and intensity of compression, but also spatial information on where the interaction takes place. Overall, these results demonstrate the dual sensing capability of the investigated TDR-based architectures and support the rationale of adapting the sensor geometry to different rehabilitation-oriented interaction modalities.

### 5.2. Feature-Based Analysis and Calibration Mapping

**Figure 12:**
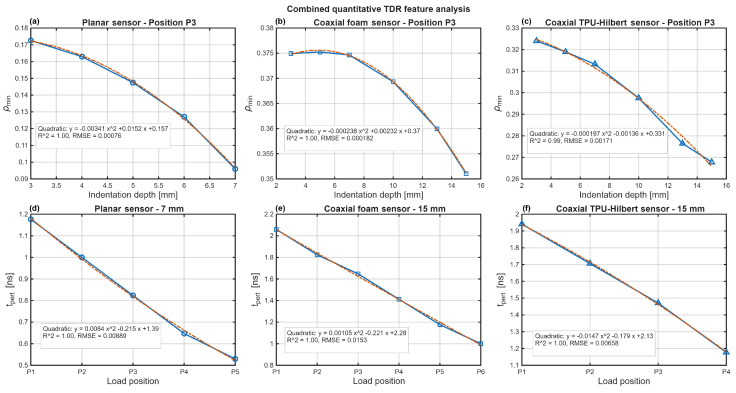
Feature-based quantitative analysis from controlled indentation tests. Panels (**a**–**c**) show $\rho_{\min}$ versus indentation depth at position P3 for the planar, foam-based coaxial, and TPU–Hilbert coaxial sensors, respectively. Panels (**d**–**f**) show $t_{\mathrm{pert}}$ versus load position at a fixed indentation depth of 7 mm for the planar sensor and 15 mm for the coaxial sensors. Dashed curves indicate second-order polynomial fits, with the corresponding $R^{2}$ and RMSE values.

Starting from the controlled mechanical measurements, a feature-based quantitative analysis was performed in order to derive preliminary calibration trends for the three sensor configurations. As aforementioned, two features were considered. The first one was the minimum value of the reflection coefficient within a selected region of interest, $\rho_{min}$, extracted at fixed load position while varying the indentation depth, and $t_{pert}$, defined as the time instant at which the local minimum of ρ(t) occurs within the selected ROI. The results of this analysis are reported in Figure 12.

The upper row shows the behavior of $\rho_{min}$ as a function of indentation depth at position P3 for the three sensor configurations. For all sensors, $\rho_{min}$ decreases as the indentation depth increases, confirming that the local amplitude of the TDR perturbation is sensitive to the applied mechanical deformation. This trend is particularly evident for the planar sensor and the TPU–Hilbert coaxial sensor, where a marked reduction in $\rho_{min}$ is observed over the investigated indentation range. The foam-based coaxial sensor also shows a decreasing trend, although the absolute variation is smaller. This behavior is consistent with the higher compliance and more distributed deformation of the foam-based structure, which can produce a less localized but still measurable modification of the reflectogram. In particular, a second-order polynomial fit was superimposed on the experimental points to provide a preliminary empirical model of the relationship between indentation depth and amplitude response. The high values of $R^{2}$ and the low RMSE values indicate that the extracted feature follows a regular trend under the investigated controlled loading conditions. These metrics describe the agreement between the experimental points and the empirical fitting curves and should not be interpreted as measures of experimental variability. Since one stabilized acquisition was retained for each position–indentation combination, statistical error bars based on repeated measurements are not reported. These fitting curves should not be interpreted as a final force calibration, since the present analysis is based on indentation depth rather than directly measured force. Nevertheless, they demonstrate that $\rho_{\min}$ can be used as a compact calibration feature for estimating the severity of the imposed deformation. The lower row of Figure 12 reports $t_{\mathrm{pert}}$ as a function of the load position, at fixed indentation depth. In this case, the temporal feature shows a clear monotonic behavior for the three sensors. This confirms the expected TDR behavior: when the mechanical perturbation is applied at different positions along the sensing element, the corresponding reflection feature appears at different time instants. Therefore, $t_{pert}$ provides information related to the spatial location of the interaction. In this perspective, the reported trends represent the first step toward the construction of calibration maps, in which the sensor response can be described as a function of both load position and indentation depth.

**Figure 13:**
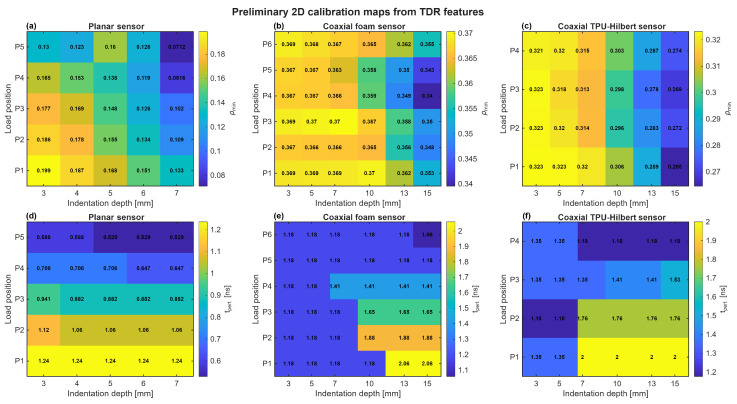
Preliminary two-dimensional calibration maps obtained from controlled mechanical indentation tests. Panels (**a**–**c**) report the $\rho_{\min}\left(P,d\right)$ maps for the planar, foam-based coaxial, and TPU–Hilbert coaxial sensors, respectively, whereas panels (**d**–**f**) report the corresponding $t_{\mathrm{pert}}\left(P,d\right)$ maps. In each panel, the color encodes the extracted feature value as a function of load position *P* and indentation depth *d*, reported on the vertical and horizontal axes, respectively. The maps provide a preliminary calibration-oriented representation of the combined deformation- and position-related responses of the three sensor architectures.

However, in a realistic interaction scenario, the contact position and the applied deformation are not necessarily known a priori and may vary simultaneously. For this reason, a two-dimensional representation of the extracted features is needed to describe the sensor response over the complete position–indentation domain. Therefore, preliminary calibration maps were constructed by extracting $\rho_{min}\left(P,d\right)$ and $t_{pert}\left(P,d\right)$ for each available combination of load position *P* and indentation depth *d*. These maps are reported in Figure 13.

The first set of three maps reports the amplitude-related feature $\rho_{min}$, whereas the second set reports the temporal feature $t_{pert}$. In these maps, the horizontal axis represents the indentation depth, the vertical axis represents the load position, and the color encodes the value of the extracted feature. The $\rho_{min}$ maps confirm that this feature is mainly sensitive to the indentation depth. Ideally, for a perfectly uniform sensor, one would expect the color to vary primarily along each row, since the indentation depth changes along the horizontal direction, while similar colors along each column would indicate a position-independent amplitude response at fixed indentation.

This behavior is particularly evident for the TPU–Hilbert coaxial sensor, where $\rho_{min}$ decreases regularly with increasing indentation depth and shows relatively similar values across different load positions. This indicates that, for this configuration, the amplitude feature is strongly associated with the degree of deformation and only moderately affected by the position of the applied load. The planar sensor also shows a clear decrease in $\rho_{min}$ with increasing indentation depth, confirming its suitability for detecting localized pressing intensity. In this case, however, the amplitude response also varies with position, as indicated by the non-uniform colors along some columns. This does not represent a limitation; rather, it confirms that the planar structure has a spatially dependent sensitivity, which is expected for a distributed TDR sensing element. In fact, the same indentation applied at different positions may produce different local impedance perturbations depending on the electromagnetic propagation path, the local mechanical deformation, and the proximity to the feeding region or sensor boundaries. The foam-based coaxial sensor shows a less pronounced variation in $\rho_{min}$ over the investigated indentation range. This behavior suggests that its response is more distributed and less sensitive to small or moderate local deformations. This result is coherent with the structural nature of the foam-based configuration, which is larger and more compliant than the other architectures. The applied indentation is likely absorbed over a wider volume, producing a weaker localized impedance perturbation. This finding also supports the rationale for introducing the TPU–Hilbert coaxial configuration, which was specifically designed to provide a more engineered and reproducible deformation response while preserving the advantages of a coaxial, grasp-oriented geometry.

The second set of Figure 13 reports the corresponding $t_{pert}$ maps. In this case, the expected behavior is different from that of $\rho_{min}$. Since $t_{pert}$ is associated with the temporal location of the TDR perturbation, it should depend mainly on the load position rather than on the indentation depth. Considering the organization of the maps, where the load position is reported on the vertical axis and the indentation depth on the horizontal axis, an ideal behavior corresponds to relatively similar colors along each row, where the position is fixed and only the indentation depth changes, and different colors along each column, where the indentation depth is fixed and the load position varies. Accordingly, horizontal color bands indicate that the temporal response is mainly governed by the load position and only weakly affected by the indentation depth.

This behavior is clearly observed for the planar sensor. For each load position, $t_{pert}$ remains relatively stable when the indentation depth changes, while a systematic variation is observed across positions. Under the considered controlled loading conditions, this indicates that the temporal feature provides a clear position-dependent response for the planar sensing structure. The stronger and more localized response of the planar configuration can be attributed to the multilayer geometry, where the conductive foam and the ground plane are close to each other and local compression directly modifies the electromagnetic boundary conditions. As a result, localized finger-like indentation produces a well-defined perturbation in the reflectogram, making the temporal feature easier to extract.

For the TPU–Hilbert coaxial sensor, the $t_{pert}$ map shows a position-dependent behavior, although less continuous than in the planar case. The presence of repeated values over some regions suggests that the temporal localization is influenced by the time resolution of the acquired reflectograms and by the more complex electromagnetic path inside the engineered coaxial structure. Nevertheless, the map still shows distinguishable temporal regions associated with different load positions, indicating that the TPU–Hilbert design improves the interpretability of the coaxial response compared with the foam-based configuration.

The foam-based coaxial sensor exhibits the least regular temporal map. Several cells show repeated $t_{pert}$ values, especially at lower indentation depths, suggesting that the local minimum detected within the selected region of interest may correspond to a common reflectogram feature rather than to a clearly position-dependent perturbation. However, at larger indentation depths, more distinct temporal values emerge, indicating that the foam-based structure becomes more informative when the applied deformation is sufficiently strong. This result is consistent with the high compliance and distributed deformation of the foam material: weak deformations may not generate a sufficiently localized impedance change, whereas stronger compression can produce a detectable position-dependent perturbation.

Overall, the calibration maps indicate that $\rho_{min}$ and $t_{pert}$ provide complementary, although not fully independent, information. While $\rho_{min}$ is mainly related to deformation intensity and $t_{pert}$ to load position, both may also be influenced by indentation depth, position, and sensor geometry. Therefore, the two features should be interpreted jointly. The maps should be regarded as a preliminary calibration framework rather than as final predictive models, as they summarize the sensing behavior of each architecture and identify the conditions under which each feature is more informative. Further validation is nevertheless required to determine whether their combined use can reliably distinguish contact position from deformation intensity under realistic loading conditions.

### 5.3. Manual Interaction Results

After the controlled characterization and the construction of preliminary calibration maps, manual interaction tests were performed to verify whether the main sensing mechanisms observed under controlled loading were also detectable during more realistic rehabilitation-inspired gestures. As aforementioned, these tests were not intended to provide a metrological estimation of force or indentation depth, since the manual deformation was not imposed by the mechanical setup. Rather, they were used as application-oriented proof-of-concept experiments and interpreted in light of the previously obtained calibration trends. The three tests were deliberately designed to reflect the specific interaction modality for which each sensor geometry is most suitable. The planar sensor was tested through localized finger pressing, since its flat extended surface is appropriate for point-like activation and target-reaching exercises. The foam-based coaxial sensor was tested under two-hand grasping, as its larger size, high compliance, and lower sensitivity to small localized variations make it more suitable for larger and more distributed compressions. Finally, the TPU–Hilbert coaxial sensor was tested during single-hand grasping, because its engineered cylindrical structure is intended to provide a more controlled and reproducible response to enclosing and circumferential squeezing actions.

**Figure 14:**
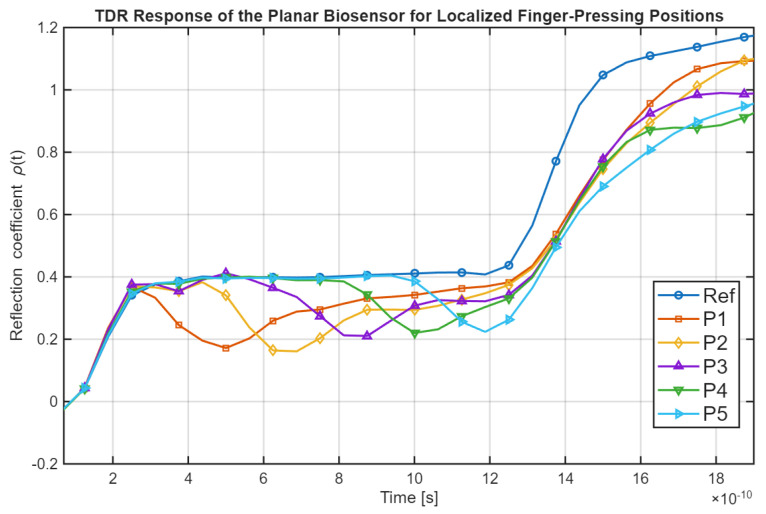
TDR response of the planar sensor during localized finger pressing at different positions. The temporal shift in the perturbation in ρ(t) confirms the ability of the sensor to discriminate the contact location.

For the planar sensor, localized finger-pressing tests were performed at predefined positions along the sensing structure. At each position, the user applied the highest comfortable pressure with one finger. A reference reflectogram was also acquired in the unloaded condition, without finger pressure, to provide a baseline response. The corresponding TDR profiles are reported in Figure 14. The reference curve represents the unloaded condition, whereas the curves P1–P5 correspond to localized finger pressing at different positions.

The manual contact produces a clear perturbation of the reflection coefficient with respect to the reference condition. More importantly, the perturbation appears at different temporal locations depending on the pressed point. This is consistent with the controlled characterization and with the calibration maps, where $t_{\mathrm{pert}}$ was shown to be mainly associated with the load position. Therefore, although the indentation depth cannot be quantitatively estimated from this manual test, the temporal displacement of the perturbation confirms the suitability of the planar configuration for spatial targeting and localized finger-pressing tasks.

**Figure 15:**
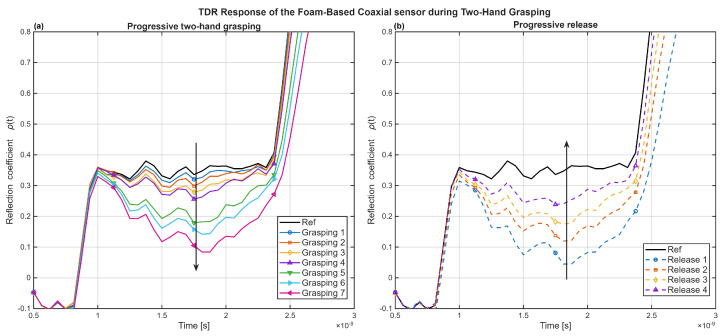
TDR response of the foam-based coaxial sensor during two-hand grasping. Panel (**a**) shows the progressive grasping phase, whereas panel (**b**) shows the progressive release phase. The curves highlight the variation in ρ(t) during bimanual compression and its partial recovery toward the unloaded reference during release. The arrows indicate the direction of the response evolution.

The foam-based coaxial sensor was tested during two-hand grasping, as shown in Figure 15. This gesture was selected because the foam-based configuration is larger and more compliant than the TPU–Hilbert sensor, and is therefore better suited to extended or bimanual interactions rather than to fine localized pressing. The left panel reports the progressive grasping phase, while the right panel shows the release phase.

During progressive bimanual compression, the reflectogram shows measurable changes in the selected temporal region, while the release phase produces a partial recovery toward the unloaded reference. This behavior is coherent with the calibration maps, where the foam-based sensor showed a more distributed response and a weaker sensitivity to small or moderate localized deformations. In particular, the $\rho_{\min}\left(P,d\right)$ map indicated limited amplitude variation over part of the indentation range, while the $t_{\mathrm{pert}}\left(P,d\right)$ map suggested that temporal localization becomes more informative mainly for larger deformations. For this reason, the foam-based coaxial sensor should not be interpreted as a lower-performance version of the TPU–Hilbert configuration, but as a complementary design more appropriate for rehabilitation tasks involving larger grasping surfaces, bilateral hand interaction, or distributed squeezing exercises.

**Figure 16:**
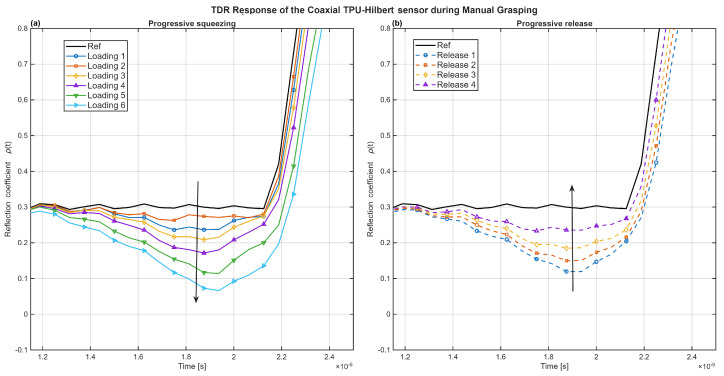
TDR response of the coaxial TPU–Hilbert sensor during manual grasping. Panel (**a**) shows the progressive squeezing phase, whereas panel (**b**) shows the progressive release phase. Progressive squeezing produces an increasing deformation-induced perturbation in ρ(t), while the release phase shows the partial recovery of the signal toward the unloaded reference condition. The arrows indicate the direction of the response evolution.

Finally, the TPU–Hilbert coaxial sensor was tested during single-hand grasping and squeezing actions. The test consisted of a loading–unloading sequence: the sensor was first grasped with low intensity, then progressively squeezed until reaching a maximum grasping condition, and finally gradually released. The corresponding TDR profiles are shown in Figure 16.

During the loading phase, the TDR response progressively changes as the squeezing intensity increases, with a more pronounced local perturbation in ρ(t). During unloading, the curves partially return toward the initial condition, indicating a reversible component of the response. This behavior agrees with the controlled calibration results, where the TPU–Hilbert configuration exhibited a regular decrease in $\rho_{\min}$ with increasing indentation depth and a more interpretable temporal response than the foam-based coaxial sensor. The engineered internal geometry likely constrains the deformation more reproducibly, leading to a clearer electromagnetic perturbation during single-hand squeezing.

Overall, the manual tests confirm the complementarity of the three proposed sensor architectures. The planar configuration is more suitable for localized finger pressing and spatial targeting; the foam-based coaxial sensor is more appropriate for large-scale distributed or two-hand grasping tasks; and the TPU–Hilbert coaxial sensor provides a more regular response to controlled single-hand squeezing. In this phase, the calibration curves and maps were used as interpretative references rather than as final predictive models. Future work will exploit these maps, together with additional TDR-derived features, to develop data-driven models capable of estimating contact position, deformation level, and interaction type from manual measurements.

## 6. Discussion

The results of this study demonstrate the potential of TDR-based deformable sensors to provide information related to both the intensity and the location of mechanical interactions relevant to motor rehabilitation. The deformation-induced modification of the local impedance appears in the time-domain reflectogram as a measurable perturbation of the reflection coefficient ρ(t). Within this framework, the amplitude-related descriptor $\rho_{\min}$ and the temporal descriptor $t_{\mathrm{pert}}$ provide complementary information, respectively associated with the deformation level and the position of the interaction. These associations should, however, be interpreted as feature trends observed under controlled loading conditions rather than as independent estimates of unknown deformation intensity and contact location.

A key outcome of the work is that the same TDR-based sensing principle can be implemented across different deformable geometries while preserving a common measurement strategy. The comparison among the three architectures shows that the TDR response is closely related to the mechanical and electromagnetic properties of each design. The planar sensor provided the clearest spatial behavior, with well-defined temporal shifts as the load position changed, making it particularly suitable for localized pressing and spatial targeting tasks. The two coaxial sensors exhibited complementary responses: the foam-based configuration showed a more distributed behavior, consistent with its larger compliant and randomly porous structure, whereas the TPU–Hilbert configuration showed a more regular and interpretable response, likely due to the engineered internal lattice. These results suggest that the coaxial geometries are better suited to grasp-oriented interactions, with the foam-based structure more appropriate for global or distributed squeezing and the TPU–Hilbert design more promising for controlled and repeatable compression.

The adopted experimental protocol also provides an initial quantitative indication of deformation and spatial discrimination. The planar sensor was tested using 1 mm indentation increments, whereas the minimum increment investigated for the coaxial sensors was 2 mm. These values represent the finest deformation sampling adopted in the present tests rather than the intrinsic resolution of the sensors. A measurable perturbation was observed at the minimum tested indentation of 3 mm for all three architectures, indicating that this deformation lies within the detectable range of the present setup, although it should not be interpreted as the formal limit of detection because lower indentation levels were not investigated. Similarly, the approximately 2 cm spacing between load positions was selected to provide a clear preliminary verification of the position-dependent temporal response and represents the spatial sampling of the present experiments rather than the ultimate spatial resolution. Future characterization using denser deformation and position sampling, repeated measurements, and background-variability assessment will allow these metrological parameters to be determined more precisely.

The present findings can also be considered in relation to recently reported rehabilitation-oriented sensing technologies. Sensorized gloves and soft robotic devices can provide detailed information on finger motion, grasping, and hand assistance, but generally require direct instrumentation of the hand and may involve multiple sensors, actuators, and dedicated calibration procedures [31,32,33,34,35]. Flexible tactile arrays provide spatially resolved pressure information through multiple sensing elements, whereas multiplexed fiber-optic systems associate deformation- or load-related responses with predefined sensing locations [41,42,43]. These technologies may offer high spatial sampling or task-specific functionality, although their implementation commonly relies on sensor arrays, predefined sensing layouts, multiple acquisition channels, or dedicated optical interrogation. The proposed TDR-based approach follows a different sensing strategy, in which deformation- and position-related information is extracted from the amplitude and temporal characteristics of a reflected perturbation using the same electrical interrogation principle across planar and coaxial geometries.

In terms of advantages and disadvantages, the main strengths of the proposed approach are the adaptability of the sensing geometry to different rehabilitation-oriented interaction modalities and the possibility of obtaining both deformation- and position-related information through a common measurement architecture. This adaptability allows the sensor configuration to be selected according to the target gesture while preserving the same TDR-based interrogation principle. The main aspects that remain dependent on the current prototype configuration are the need to calibrate each architecture according to its geometry and intended loading conditions and to further consolidate the simultaneous interpretation of position and deformation intensity under previously unseen and more realistic interactions. These aspects are consistent with the preliminary calibration-oriented nature of the present study and can be systematically addressed through extended calibration and validation procedures.

As shown by the feature-based analysis, the one-dimensional trends and two-dimensional maps already provide an initial calibration-oriented representation of the response of each architecture. One-dimensional curves are useful for highlighting specific trends, such as the variation in $\rho_{\min}$ with indentation depth or the shift in $t_{\mathrm{pert}}$ with load position. However, realistic manual interactions involve the simultaneous variation in contact position and deformation level. The two-dimensional feature maps therefore provide a more complete calibration-oriented representation of the tested position–indentation space, supporting the comparison among architectures and helping to identify the conditions under which each descriptor is most informative. Accordingly, they provide a preliminary framework for the joint interpretation of amplitude- and time-related information. Since both descriptors may be influenced by the simultaneous variation in position, indentation depth, and sensor geometry, their combined interpretation is more appropriate than considering either feature as an independent estimator. The present results characterize the response patterns associated with the investigated loading combinations and provide the basis for extending the calibration framework toward the robust assessment of unknown position and deformation conditions when they vary simultaneously.

Taken together, the controlled mechanical tests, feature-based maps, and manual interaction trials provide consistent experimental support for the proposed sensing concept. The results show that the investigated TDR-based sensors are not only able to detect mechanical deformation, but can also provide information related to where the interaction occurs, with a clarity that depends on the sensor architecture and loading condition. In addition, the manual tests showed response patterns consistent with those observed under controlled conditions during representative rehabilitation-oriented gestures. The present study therefore provides a structured basis for the development of geometry-adaptable sensing platforms for rehabilitation monitoring.

## 7. Conclusions and Future Work

This work investigated three deformable TDR-based sensor architectures for the monitoring of rehabilitation-inspired mechanical interactions. The results showed that deformation-induced variations in the time-domain reflection coefficient ρ(t) can provide information related to both interaction intensity and contact location under controlled loading conditions. The comparison among the three architectures confirmed that the sensor geometry plays a key role in determining the most suitable interaction modality. The planar sensor exhibited a clear spatial response and appears suitable for localized finger pressing and spatial targeting tasks. The foam-based coaxial sensor exhibited a more distributed behavior, coherent with larger two-hand grasping and squeezing interactions. The TPU–Hilbert coaxial sensor provided a more regular and interpretable behavior, making it promising for controlled single-hand squeezing tasks.

The feature-based analysis provided a preliminary calibration-oriented representation of the combined effects of load position and deformation level across the investigated sensor architectures. Within the tested conditions, the proposed approach enabled the extraction of position- and intensity-related information, although further validation is required before the two quantities can be robustly estimated when they vary simultaneously.

Future work will focus on extending the experimental validation through additional positions, deformation levels, repeated measurements, different users, and direct force acquisition. Further analyses will systematically address repeatability, hysteresis, durability, sensor-to-sensor variability, and long-term stability. A denser and more structured calibration dataset will then be used to investigate multivariable calibration procedures based on the combined response of $\rho_{\min}$ and $t_{\mathrm{pert}}$, with the aim of more robustly distinguishing contact position from deformation intensity when both vary simultaneously. The resulting calibration models will be trained and evaluated using independent measurements and under more realistic rehabilitation-related loading conditions.

Machine learning methods may further support this calibration process by identifying response patterns that are not fully represented by individual features. Once sufficiently extensive datasets are available, supervised classification and regression models could jointly analyze $\rho_{\min}$, $t_{\mathrm{pert}}$, and additional descriptors extracted from the complete reflectogram to estimate loading conditions, recognize different interaction modalities, and account for variability among sensor architectures and users [44].

Future investigations will also examine the influence of sensor dimensions and internal geometry on both the electromagnetic and mechanical response. In particular, coaxial configurations with different conductor diameters, radial dimensions, and dielectric structures will be considered to assess the trade-off among characteristic impedance, mechanical compliance, required interaction force, and TDR sensitivity. Manufacturing tolerances and surface finishing will also be considered to assess their possible influence on response repeatability and calibration consistency. In parallel, alternative solutions for the external conductive layer, including a silver-particle-based conductive coating in place of the currently employed conductive adhesive film, will be investigated to improve adhesion to the deformable substrate, conformity at the body interface, mechanical flexibility, and electrical-contact stability during deformation. These analyses will support a more systematic optimization of the sensor geometry, material interfaces, and fabrication process according to the target rehabilitation gesture and interaction modality.

The deformability of the proposed sensors may also support future wearable implementations, particularly through the integration of the planar architecture into rehabilitation gloves, soft bands, or other body-conformable supports [45]. Such developments would require miniaturized interrogation electronics and further evaluation of comfort and signal stability during movement.

In addition, future sensor architectures may include integrated inertial units, such as accelerometers, to combine TDR-derived interaction information with movement-related parameters. This integration could enable the simultaneous assessment of the mechanical interaction with the rehabilitation device and the monitoring of involuntary motor components, such as tremor in patients with Parkinsonian symptoms, during the execution of therapeutic gestures.

## Data Availability

The data presented in this study are available from the corresponding authors upon reasonable request.

## Abbreviations

The following abbreviations are used in this manuscript:

TDR	Time-Domain Reflectometry
EM	Electromagnetic
SUT	System Under Test
SE	Sensing Element
VNA	Vector Network Analyzer
ROI	Region of Interest
TPU	Thermoplastic Polyurethane
PTFE	Polytetrafluoroethylene
RMSE	Root Mean Square Error
